# Ultra-high modulation depth exceeding 2,400% in optically controlled topological surface plasmons

**DOI:** 10.1038/ncomms9814

**Published:** 2015-10-30

**Authors:** Sangwan Sim, Houk Jang, Nikesh Koirala, Matthew Brahlek, Jisoo Moon, Ji Ho Sung, Jun Park, Soonyoung Cha, Seongshik Oh, Moon-Ho Jo, Jong-Hyun Ahn, Hyunyong Choi

**Affiliations:** 1 School of Electrical and Electronic Engineering, Yonsei University, Seoul 120-749, Korea; 2 Department of Physics and Astronomy, Rutgers, The State University of New Jersey, Piscataway, New Jersey 08854, USA; 3 Center for Artificial Low Dimensional Electronic Systems, Institute for Basic Science (IBS), Pohang University of Science and Technology (POSTECH), 77 Cheongam-Ro, Pohang 790-784, Korea; 4 Division of Advanced Materials Science, Pohang University of Science and Technology (POSTECH), 77 Cheongam-Ro, Pohang 790-784, Korea; 5 Institute for Advanced Materials, Devices and Nanotechnology, Rutgers, The State University of New Jersey, Piscataway, New Jersey 08854, USA; 6 Department of Materials Science and Engineering, Pohang University of Science and Technology (POSTECH), 77 Cheongam-Ro, Pohang 790-784, Korea

## Abstract

Modulating light via coherent charge oscillations in solids is the subject of intense research topics in opto-plasmonics. Although a variety of methods are proposed to increase such modulation efficiency, one central challenge is to achieve a high modulation depth (defined by a ratio of extinction with/without light) under small photon-flux injection, which becomes a fundamental trade-off issue both in metals and semiconductors. Here, by fabricating simple micro-ribbon arrays of topological insulator Bi_2_Se_3_, we report an unprecedentedly large modulation depth of 2,400% at 1.5 THz with very low optical fluence of 45 μJ cm^−2^. This was possible, first because the extinction spectrum is nearly zero due to the Fano-like plasmon–phonon-destructive interference, thereby contributing an extremely small denominator to the extinction ratio. Second, the numerator of the extinction ratio is markedly increased due to the photoinduced formation of massive two-dimensional electron gas below the topological surface states, which is another contributor to the ultra-high modulation depth.

## Abstract (web summary)

For optical control of plasmons metals require a large amount of power in the control pulse, yielding a small modulation depth. Here, Sim *et al.* fabricate arrays of Bi_2_Se_3_ and report a modulation depth of 2,400% at 1.5 THz with an optical fluence of 45 μJ/cm^2^, demonstrating a novel route for controlling plasmons.

## Graphical abstract

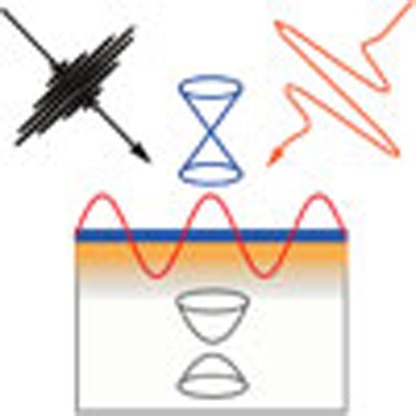

Ultrafast optical control of plasmon provides a potential route towards novel high-speed active photonic devices^1^,^2^,^3^,^4^,^5^,^6^,^7^,^8^,^9^,^10^,^11^,^12^. For this reason, considerable efforts have been devoted to finding the best material for controlling plasmons. Conventionally, noble metals have been regarded as representative plasmonic materials, because there exist plenty of charges for the collective excitations. However, optical controllability of plasmons in metals is significantly lower due to the small modulation depth and requirement of large amount of power from the control pulse^5^,^13^. In contrast, the plasmonic response in semiconductors can be easily modulated by the optical pulse due to creation of large number of photo-generated carriers. For example, a recent measurement on narrow-gap semiconductor indium arsenide (InAs) demonstrated a very high plasmonic modulation depth^5^. Despite such improvement, optical modulation of plasmons in semiconductors is still limited by the photo-generated carrier density, which means that a powerful laser source is inevitably necessary to achieve a higher modulation depth. Thus, semiconductor-based plasmonics inherently requires operating only in the strongly photoexcited regime, which significantly restricts the optoelectronic functionalities.

Three-dimensional topological insulators (TIs) are novel electronic systems, where semiconductor-like states in bulk coexist with metallic Dirac states on surfaces^14^,^15^,^16^,^17^,^18^,^19^, as illustrated in Fig. 1a. Unlike conventional heterostructure systems, the metal-semiconductor coexistence is naturally formed in the TIs without hetero-epitaxial growth or artificial stacking of two dissimilar phases of materials. Thus, it should be an ideal material for achieving ultrafast active plasmonics, because both metallic and semiconducting behaviours can be fully exploited. Recent works have demonstrated that the surface Dirac plasmons (red line in Fig. 1a) can be excited in the metallic topological surface state (TSS) in the terahertz (THz) and optical range^20^,^21^,^22^,^23^,^24^. Especially, Di Pietro *et al.*^20^ demonstrated the frequency-tunable topological Dirac plasmons, which exhibit outstanding robustness of the TSS against temperature variations. Furthermore, as illustrated in the left panel of Fig. 1b, they found that the unique spectral lineshape arises from the interaction of plasmon with a 1.9-THz phonon, which provides a potential degree of freedom for realizing the optically tunable active plasmonics. No experimental studies exist to investigate such ultrafast controllability of plasmons in TIs. More importantly, it remains quite elusive how the large optical absorption in semiconductor-like bulk would affect the surface plasmon and the plasmon–phonon interaction.

In this work, we explore the ultrafast optical modulation of plasmons in a micro-ribbon array of TI Bi_2_Se_3_ by using time-resolved optical pump THz-probe spectroscopy. Under optical modulation pulse injection (see Fig. 1b), the surface plasmon frequency is shifted beyond the phonon frequency in the THz range, which significantly changes the extinction spectra. As a result, an unprecedented, giant modulation depth up to 2,400% is obtained with very low fluence of optical control pulse (45 μJ cm^−2^). Our theoretical calculations show that the plasmon frequency shift arises from the photo-doping of the non-topological two-dimensional electron gas^25^ (2DEG) formed due to downward bending of the bulk bands near the surface. Unlike conventional semiconductor-based plasmonics, various species of quantum states, such as Dirac electrons, massive 2DEG and semiconductor-like bulk, which together with plasmon–phonon interference lead to dynamic spectral modulation in TIs, provide a novel platform for controlling plasmons.

## Results

### Plasmon response without control pulse injection

**Figure 2:**
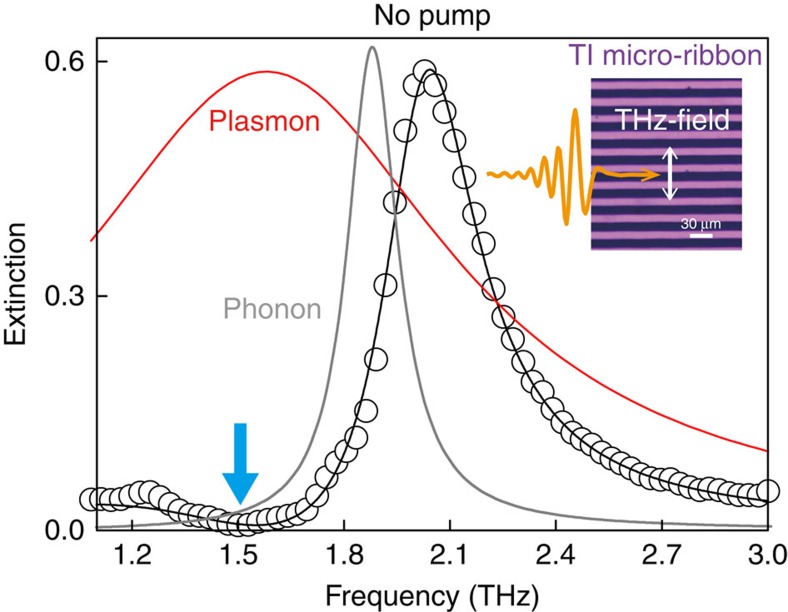
Extinction spectrum of Bi_2_Se_3_ micro-ribbon without control pulse injection. Normalized bare plasmon resonance (red line) is extracted from the measured extinction spectrum (black circles) through a plasmon–phonon interaction model fit (black line). The non-interacting phonon resonance (grey line) is extracted from measuring the extinction spectrum with THz polarization parallel to the micro-ribbon axis (see Supplementary Note 1, Supplementary Figure 1 and Supplementary Table 2). The blue arrow near 1.5 THz indicates spectral dip arising from the Fano-interference of plasmon with phonon. (Inset): optical image of Bi_2_Se_3_ micro-ribbon. THz probe field (yellow line) is vertically incident on the ribbon plane, whose polarization is perpendicular to the ribbon axis, as indicated by a white arrow.

Before exploring the ultrafast optical modulation of plasmons in the TI, it is instructive to understand the microscopic origin of the spectral response without the optical control pulse. Figure 2 shows the THz extinction spectra of our Bi_2_Se_3_ micro-ribbon thin-film array with thickness of 100 quintuple layers (QLs; 1 QL≈1 nm) (see inset of Fig. 2 for the sample image and Methods for experimental details and sample preparation). The ribbon-width *W* and array period 2*W* are chosen as 15 and 30 μm, respectively, to locate the plasmon frequency in our spectral window of 1.1–3 THz, which is based on the calculation of the plasmon frequency (see below). The measured spectrum closely resembles a prior work^20^, showing a dip near 1.5 THz (indicated by a blue arrow) and a peak at 2.1 THz. Following Di Pietro *et al.*^20^, we understand this response as a result of the Fano-type interference between the relatively broad plasmon response and the narrow phonon peak, as revealed by the fit (black solid line) of the plasmon–phonon interaction model to the data (see Methods for details of the plasmon–phonon interaction model). From the fitting analysis, we can extract the bare plasmon response centred at 1.58 THz (red curve) and the phonon frequency of 1.87 THz (grey curve), both of which agree well with the previous measurements^20^,^23^ (all fitting parameters are presented in Supplementary Table 1). For further justification of our analysis, we compare the measured plasmon frequency with the following theoretical model developed by Stauber *et al.*^26^,^27^,


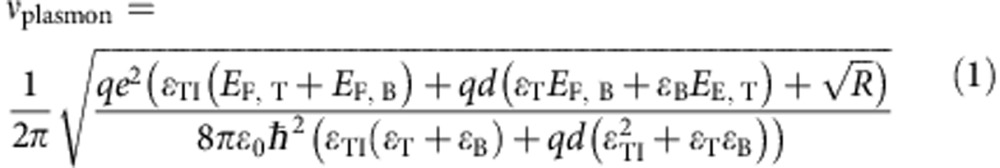


with


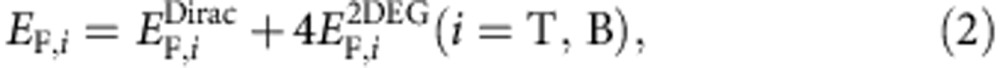


where 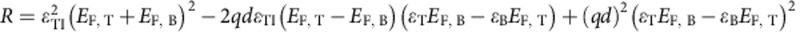, *v*_plasmon_ is the bare plasmon frequency, *q*∼*π*/*W*=2.094 cm^−1^ is the plasmon wavevector, subscripts T and B indicate the top and bottom surface, ɛ_T_=1 and ɛ_B_=10 are the dielectric constants at surfaces and ɛ_TI_=100 is the dielectric constant at the centre. A notable fact, compared with the prior work^20^, is that Fermi levels of both Dirac electrons 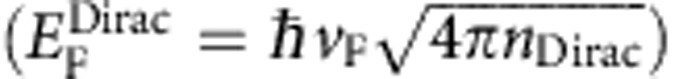 and 2DEG 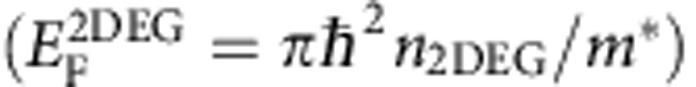 contribute to the surface plasmon excitation (see equation (2))^26^,^27^; here, *v*_F_=6±1 × 10^5^ m s^−1^ is the Dirac Fermi velocity^20^, *n*_Dirac_∼1.5±0.5 × 10^13^ cm^−2^ (*n*_2DEG_∼3.75±1.75 × 10^12^ cm^−2^) is the two-dimensional carrier density of Dirac (2DEG) state per surface and m*=0.15±0.01*m*_0_ is the effective mass of the 2DEG electrons^20^. The pre-factor ‘4' of 
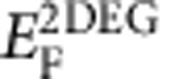
 in equation (2) originates from the spin-degeneracy and the quadratic energy-momentum dispersion of the 2DEG states (see Supplementary Note 3 for further discussion)^26^,^27^. Equations (1) and (2) yield *v*_plasmon_∼1.61 THz, which agrees extremely well with the measured value of 1.58 THz. Here, the contribution of Dirac states 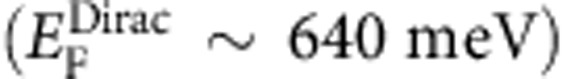 to plasmonic excitation is approximately twice as large as that of 2DEG 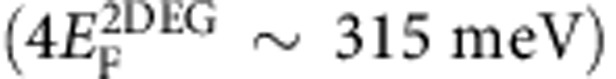, revealing the topological nature of plasmon excitation.

### Ultra-high modulation depth of topological surface plasmon

With this understanding of the extinction spectrum, we present the optical modulation of plasmonic response in the TI. The modulation depth of probe extinction is defined as follows^5^,^10^,^28^,^29^:


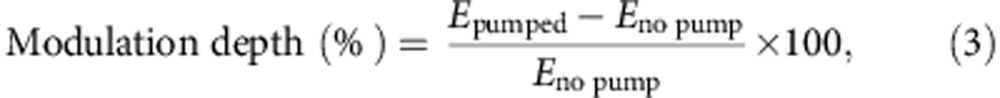


where *E*_pumped_ (*E*_no pump_) is extinction coefficient with (without) the optical pump injection. It is clear from the equation (3) that higher differential extinction, *E*_pumped_−*E*_no pump_, leads to a larger modulation depth. Here, we emphasize that the small denominator *E*_no pump_ can also lead to a large modulation depth, which has been usually overlooked in the fields of the ultrafast active plasmonics. In this work, the extinction coefficient of Bi_2_Se_3_ (*E*_no pump_) is extremely low near 1.5 THz due to the Fano-type interference of Dirac plasmons with the THz bulk phonon (see blue arrow in Fig. 2). Thus, we can expect a giant modulation depth if the optical control pulse gives rise to an increase in the extinction coefficient in this transparent region.

**Figure 3:**
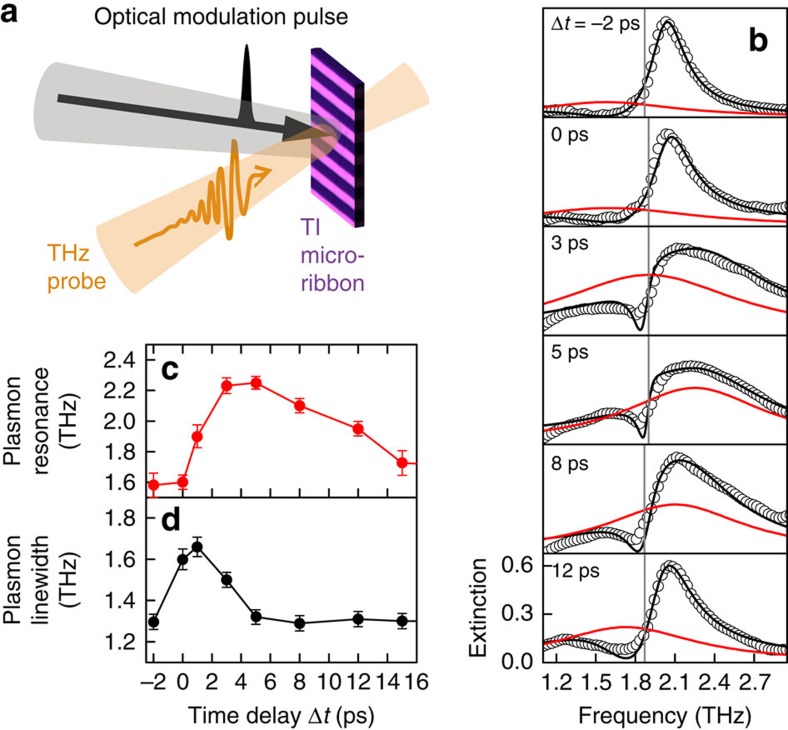
Ultrafast plasmon modulation by injecting control pulse. (**a)** Schematic illustration of time-resolved optical pump terahertz-probe spectroscopy. Polarizations of optical modulation pump (1.55 eV, 50 fs) and THz probe are 45° and perpendicular to the ribbon axis, respectively. (**b**) Transient extinction spectra (black circles) are displayed for several pump–probe time delays (Δ*t*), measured at 78 K with 45 μJ cm^−2^ of optical excitation fluence. Red lines and grey vertical lines indicate the bare plasmon resonance and the phonon frequency, respectively, which are extracted from the plasmon–phonon interaction model (black lines). (**c**,**d**) Time-resolved dynamics of plasmon resonance frequency (**c**) and linewidth (**d**). The error bars represent 95% confidence intervals for the fitting parameters.

To verify the above rationale, we performed the time-resolved, optical pump THz-probe spectroscopy (see Fig. 3a). The 1.55-eV, 50-fs optical pulse with fluence of 45 μJ cm^−2^ serves as a control pulse, whose polarization is at 45° with respect to the ribbon axis. We keep the THz probe-field polarization perpendicular to the ribbon axis for the plasmon excitation. We first discuss the overall feature of the time-resolved THz dynamics under optical pulse injection. Figure 3b shows the measured transient extinction spectra (black dots) and the fits using the plasmon–phonon interaction model (black line) for several pump–probe time delays (Δ*t*). Corresponding bare plasmon responses (red lines) and phonon frequencies (vertical grey line) are also displayed (see Supplementary Note 1 for all fitting parameters and related discussions). We see that the plasmon resonance is largely blue-shifted beyond the phonon frequency at Δ*t*=5 ps, giving rise to the characteristic changes in the transient extinction lineshapes, where the increased spectral amplitudes near 1.5 THz and 2.2–2.5 THz are clearly visible. The transient plasmon frequencies and linewidths are displayed in Fig. 3c,d, respectively. The time-dependent dynamics of these two parameters exhibit very different timescales; the maximum plasmon frequency occurs at Δ*t*=5 ps, while the linewidth has already recovered to the unexcited value by that time. Such behaviour provides a practical merit, because the plasmon can be shifted without broadening even under optical injection, suggesting that the efficient spectral modulation is possible under non-equilibrium condition. By comparing the THz dynamics of the micro-ribbon arrays with that of unpatterned Bi_2_Se_3_ samples, we found that the transient plasmon frequencies follow the dynamics of the photo-generated carrier population, while the linewidths follow the trend of effective surface electronic temperature (see Supplementary Note 2 and Supplementary Figure 2 and 3 for a detailed discussion on this issue).

**Figure 4:**
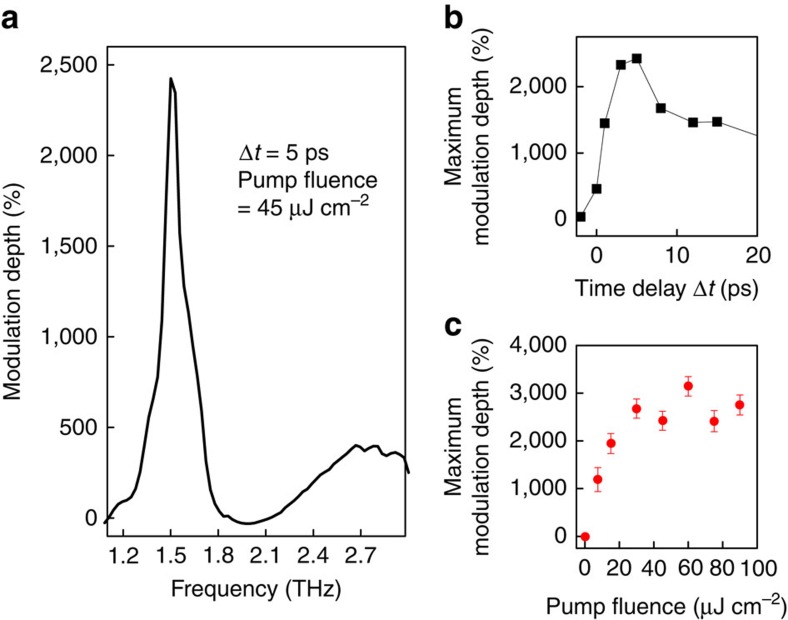
Ultra-high modulation depth of extinction coefficient exceeding 2,400%. (**a**) Spectrally resolved transient modulation depth at Δ*t*=5 ps. (**b**) Dynamics of the maximum modulation depth with optical excitation fluence of 45 μJ cm^−2^. (**c**) Fluence-dependent maximum modulation depth. The corresponding extinction spectra are shown in Fig. 6b. The error bars represent 95% confidence intervals estimated by taking multiple measurements.

Our key experimental observation is shown in Fig. 4a. There, we obtained an extremely large modulation depth of 2,400%. This value by far exceeds the recent record value of 1,100% observed in the narrow-gap InAs semiconductor^5^. Furthermore, the fluence of the control pulse is only half of that in ref. 5, indicating exceptional optical modulation in TI systems. It is obvious that the shifted plasmon resonance leads to the large spectral modulation, because the largest modulation depth occurs simultaneously when the plasmon reaches its maximum shift at Δ*t*=5 ps (see Figs 3c and 4b). More importantly, we note that the maximum spectral modulation takes place at 1.5 THz, at which the extinction coefficient without optical pump injection (*E*_no pump_) exhibits almost a transparent behaviour due to the Fano-like interference of Dirac plasmons (see again the blue arrow in Fig. 2). Figure 4c shows the fluence dependent modulation depth, in which the demodulation depth can easily exceed 2,400% with increasing the control pump fluence. The other modulation peak near 2.7 THz (Fig. 4a) was observed, but the modulation depth is relatively small because of the large non-zero magnitude of *E*_no pump_. These results clearly indicate that the extremely small denominator (*E*_no pump_) in equation (3) plays an important role in determining the modulation depth near 1.5 THz. In fact, this distinguishes the TI systems from other conventional semiconductors, where the collective oscillation of Dirac electrons in TI systems gives rise to the transparent frequency region via Fano-interference with the bulk phonon, even without the optical excitation.

### Plasmon modulation at room temperature

One of important benefits of the Dirac plasmons in TIs is the robustness of the TSSs against temperature variations, which was confirmed by a recent study^20^; the extinction lineshape of the TSS plasmon remains virtually unchanged in the range of 6–300 K. This is one distinct characteristics of TIs compared with other non-topological plasmonic materials^20^,^24^. In the above section, we have discussed the unprecedented large plasmonic modulation depth at 78 K. With considering the robust TSS against temperature, one can expect that the modulation depth will still be large at room temperature.

**Figure 5:**
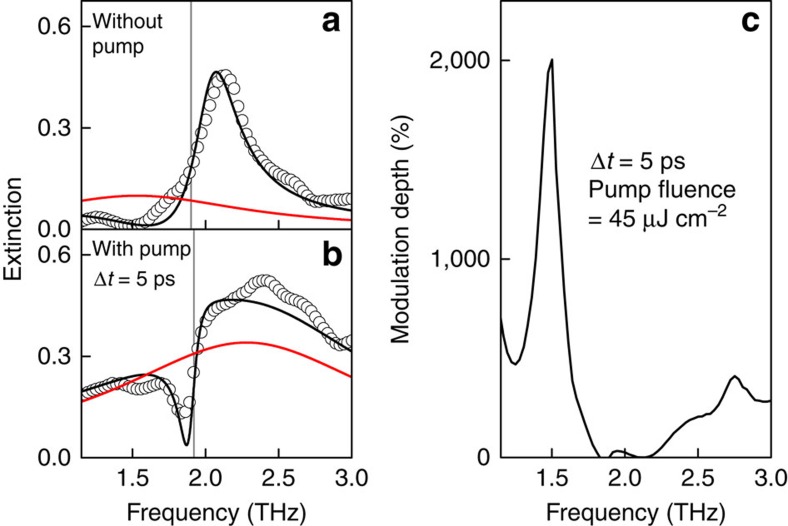
Plasmon modulation at room temperature (300 K). (**a**) Extinction spectrum without control pulse injection (black circles). (**b**) Transient extinction spectrum (black circles) at Δ*t*=5 ps, under optical excitation with optical pump fluence of 45 μJ cm^−2^. The red and grey vertical line is the bare plasmon resonance and the phonon frequency, respectively. (**c**) Corresponding modulation depth at Δ*t*=5 ps is shown.

We performed the same experiment at 300 K without changing any experimental conditions. Figure 5 displays the extinction spectra and the corresponding modulation depth at Δ*t*=5 ps. For the reference spectrum without photoexcitation (Fig. 5a), the lineshape closely resembles the one measured at 78 K (Fig. 2), as expected. In particular, the spectral dip at 1.5 THz is still observable owing to the robustness of TSS. This temperature-insensitive property of Dirac plasmon provides an experimental testimony for the high modulation depth at room temperature. For the extinction spectrum under control pulse excitation (Fig. 5b), the plasmon centre is shifted to a higher frequency (red line). Notable is that the amount of plasmon shift is almost unchanged even though the temperature is increased (the origin of the pump-induced plasmon shift is discussed in the next section). This, together with the spectral dip at 1.5 THz in Fig. 5a, gives rise to the large modulation depth, as shown in Fig. 5c, where the maximum modulation depth (∼2,000%) is still much larger than other non-topological materials^3^,^4^,^5^,^10^. Note that this value is somewhat smaller than the data at 78 K (∼2,400%). This is because the background scattering is increased (see Supplementary Tables 1 and 3), and consequently the denominator of equation (3) (*E*_no pump_) is also increased.

### Origin of the pump-induced plasmon shift

**Figure 6:**
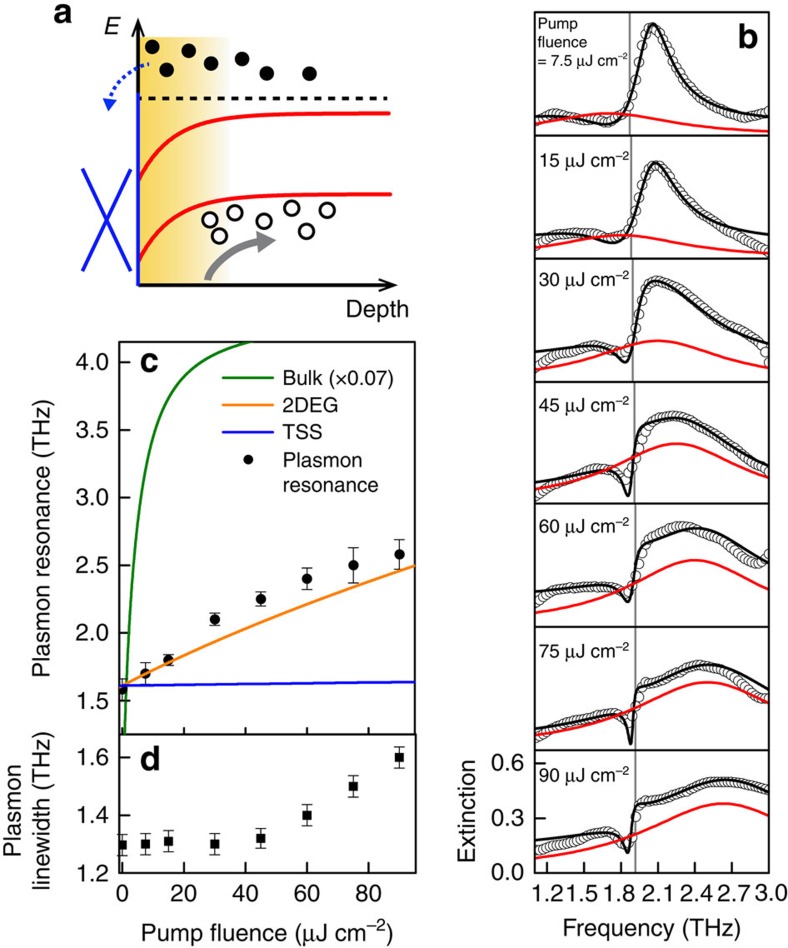
Origin of the pump-induced plasmon frequency shift. (**a**) Energy band diagram in real space is shown. Upon photoexcitation, although some of the photo-generated electrons (black-filled circles) in bulk are scattered into TSS (dashed blue arrow), the amount of increased density of Dirac electrons is too small to cause the measured plasmon frequency shift. Instead, photo-doping of 2DEG (yellow-shaded region) is responsible for the increase of the plasmon frequency. Photo-generated holes (black open circles) are assumed to be drifted into the bulk due to downward band-bending (red lines), as indicated by the grey arrow. (**b**) Pump-fluence-dependent extinction spectra (black circles) at 78 K for a fixed pump–probe time delay at Δ*t*=5 ps are displayed with the plasmon–phonon Fano fits (black lines). Red lines and grey vertical lines indicate the bare plasmon resonance and the phonon frequency, respectively. (**c**,**d**) Corresponding plasmon frequency (black circles (**c**)) and linewidth (black squares (**d**)) are shown. Solid lines in **c** show the theoretically calculated pump-fluence dependence of plasmon frequency without using any free-fitting parameters. The error bars represent 95% confidence intervals for the fitting parameters. While the increased 2DEG density under photoexcitation well reproduces the measured plasmon frequency shift (yellow line) via equation (1), contribution from the increased TSS electron density to the plasmon (blue line) is too small, compared with the measured data. Surface plasmon excitation of unconfined three-dimensional bulk carriers (green line, scaled by a factor of 0.07) is by far beyond the frequency window in this measurement.

To understand more details on the optical modulation of the plasmonic response in TIs, we now discuss the origin of the plasmon shift. We first consider the contribution of pump-induced change in Dirac plasmons. A recent study in graphene reported that the frequency of Dirac plasmons is blue-shifted immediately after photoexcitation, due to increased effective electronic temperature, and the corresponding plasmon linewidth also showed large broadening with the same timescale under transient excitation conditions^10^. In TI systems, however, the maximum plasmon frequency shift emerges after the linewidth of the plasmon recovers from the transient maximum, as shown in Fig. 3c,d. We therefore rule out the effect of electronic temperature on the plasmon shift. Next, we consider the possible contribution from photo-doping of TSS to the change in the plasmon frequency. It is well known that, upon photoexcitation, electron population of TSS increases due to scattering of the photo-generated electrons from the bulk, as indicated by a dashed blue arrow in Fig. 6a. To understand how it affect the plasmon shift, we carried out pump-fluence-dependent extinction measurements and compared the data with theoretical calculations. Figure 6b displays the extinction spectra with varying pump fluence at Δ*t*=5 ps. Bare plasmon responses (red lines) are extracted from the fits of the plasmon–phonon interaction model (black lines), and the corresponding centre frequencies and linewidths are plotted in Fig. 6c,d, respectively. We theoretically obtained the plasmon resonance frequency using equations (1) and (2), assuming that the optical excitation increases only the top-surface Dirac Fermi level 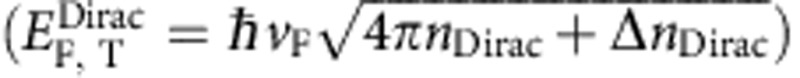. This is reasonable because the penetration depth (∼20 nm) of the optical pulse is much shorter than the sample thickness (∼100 nm)^30^, where 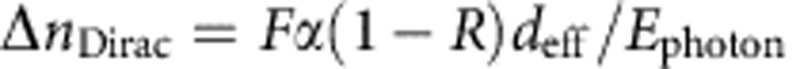 is the pump-generated Dirac electron density (*F* is the pump fluence, *α*=5 × 10^5^ cm^−1^ is the absorption coefficient^30^, *R*∼0.5 is the reflectivity, *d*_eff_=0.4 QL is the effective number of bulk layers contributing to the increase in Dirac electron density^31^, *E*_photon_=1.55 eV is the pump photon energy). As shown in Fig. 6c, the calculated plasmon frequency (blue line) is much lower than the measured data (black circles). Therefore, it is highly likely that the photo-doping of TSS is too low to reproduce the measured plasmon shift.

A second possibility is that the pump-induced frequency shift of plasmon originates from the increase in 2DEG electron density. To verify this, we calculate the plasmon frequency again by using equations (1) and (2), assuming that photo-generated electrons in the band-bending region (yellow-shaded region in Fig. 6a) relax to the two-dimensional confined states, and subsequently increase the 2DEG Fermi level 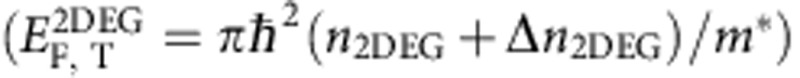 in the TSS, where 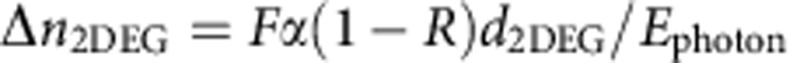 is the increased 2DEG density and *d*_2DEG_∼4 QL is the 2DEG depth^32^. Here, we neglect the pump-generated holes because they drift to the deeper bulk region due to downward band-bending (see a grey arrow in Fig. 6a)^33^. The calculated plasmon frequency (yellow line) agrees well with the measured data without using any free-fitting parameters, as shown in Fig. 6c, which seemingly reproduces the pump-induced plasmon shift. We note that the measured plasmon linewidth is largely increased at *F*≥50 μJ cm^−2^ (Fig. 6d). This may be attributed to the uncooled hot electron at the time delay of Δ*t*=5 ps, because it directly reflects the electron temperature (see Supplementary Note 4 for more detailed discussions about transient plasmon damping).

The above analysis assumes that the two-dimensionally confined carriers (TSS and 2DEG) are largely responsible for the observed optical modulation of plasmons. For completeness, we have also considered the possibility that the observed plasmon resonance originates from the conventional surface-charge oscillation of the unconfined, three-dimensional bulk electrons^34^. As shown in Fig. 6c, the calculated frequency of this plasmon mode^35^ 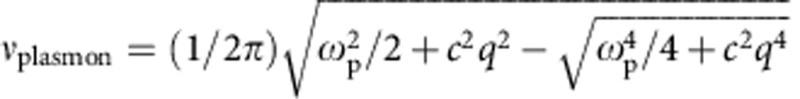 largely exceeds our THz spectral window (green line, scaled by a factor of 0.07), where 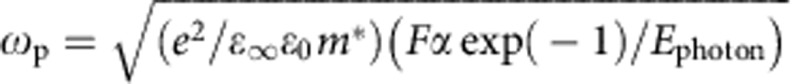 is the bulk plasma frequency of pump-generated electrons within the photoexcited region. Thus, we exclude the contribution of three-dimensional carriers to the measured plasmonic resonance (see Supplementary Note 5 for a more detailed discussion).

## Discussion

What distinguishes TI, compared with conventional plasmonic systems, is that various species of quantum states coexist, namely TSS, 2DEG and normal semiconductor-like bulk, all of which play a crucial role in modulating the plasmonic response. First, owing to the TSS, the Dirac plasmon can be excited without optical injection, providing a transparent spectral region for efficient modulation of the extinction coefficient via the Fano-type interference with a narrow phonon mode. Second, 2DEG states are responsible for the pump-induced shift of the plasmon resonance, which dominantly contribute to the modulation of the extinction spectra. Third, the semiconductor-like bulk state behaves as a photoelectron reservoir^36^ for the two-dimensional surface states under optical excitation, leading to the efficient plasmon shift. Owing to the contributions from all of these states, TI exhibits an unprecedentedly giant modulation depth of 2,400% with very low pump fluence (45 μJ cm^−2^). We more clearly see the advantage of TI by comparing the performance with other plasmonic systems. Following Wagner *et al.*^5^, we show the figure of merit (the ratio of modulation depth to the fluence of optical control pulse) for various active plasmonic systems in Table 1: the giant figure of merit in TI (∼50,000) by far surpasses the recent, record-high value observed in the narrow-gap semiconductor InAs (∼18,000, ref. 5) and other materials^3^,^4^,^5^,^10^,^11^, as well. For the transient dynamics, TI shows a somewhat slow response (orders of 10–100 ps)^31^,^33^,^36^,^37^,^38^,^39^,^40^,^41^,^42^. However, considering that the defect engineering^5^ can reduce the response time in general, we expect that faster plasmonic TI system can be implemented if desired.

Looking towards the future, this TI-based active plasmonic system may open up a novel route towards optically controlled ultra-high contrast plasmonic switches^2^,^3^,^4^,^5^,^8^,^10^,^11^. When designing the devices, it is important to note that this system is highly frequency-selective. Thus, depending on the type of applications, one should carefully choose the micro-ribbon spacing, which determines the fundamental plasmon frequency^20^.

## Methods

### Sample preparation

Bi_2_Se_3_ thin films were grown on Al_2_O_3_ (0001) substrates using a custom-made SVTA MOSV-2 MBE system with base pressure less than 3 × 10^−10^ Torr. Bismuth and selenium sources with 99.999% purity were thermally evaporated from Knusden cells to provide stable fluxes, which were measured using an Inficon BDS-250 XTC/3 Quartz crystal microbalance (QCM) system. The kSA 400 reflection high-energy electron diffraction measurement system was used to monitor the crystal quality of Bi_2_Se_3_ thin-films during growth.

The substrates were cleaned *ex situ* by placing them in a ultraviolet/ozone cleaner for 5 min to remove organic contaminants on the surface. Immediately afterwards, they loaded into the MBE growth chamber. In the growth chamber, the substrates were heated to 750 °C for 10 min in 1 × 10^−6^ Torr oxygen environment to remove any excess contaminants. Bi_2_Se_3_ thin films were then grown by employing a two-step growth procedure developed at Rutgers University as reported by Bansal *et al.*^43^ Initially, a seed layer of 3 QL (1 QL∼1 nm) Bi_2_Se_3_ was grown at 135 °C followed by annealing to 300 °C for 10 min before depositing rest of the film at 300 °C. During the entire growth, Se:Bi flux ratio of ∼10 was maintained. Film thickness was determined *in situ* by QCM and *ex situ* by Rutherford back-scattering. High quality of the growing film was indicated by a sharp reflection high-energy electron diffraction pattern and associated Kikuchi lines.

The 15-μm-width line pattern with 1:1 ratio gap among the line was formed using AZ1512 (1.2 μm in thickness) followed by a hard-baking process at 100 °C for 10 min, and dry etching process using reactive ion etcher (RIE, SF6, 40 s.c.c.m., 140  torr and 100 watt). The etching rate was evaluated as 27 QL per min. The photoresist was clearly stripped off by being brushed in acetone.

### THz spectroscopy

The THz^44^ spectral measurements were performed based on a 250-KHz Ti:sapphire-regenerative amplifier system (Coherent RegA 9050). The 1.55-eV (50 fs) pulses serve to generate THz pulses via optical rectification and to detect them through electro-optic sampling in a pair of <110> ZnTe crystal. The sample was maintained at the pressure of 7 × 10^−6^ Torr in a cryostat during all measurements. To characterize the extinction spectra of the Bi_2_Se_3_ micro-ribbon, we employed THz time-domain spectroscopy in transmission geometry without optical pump injection. As described in ref. 45, the extinction spectrum is determined by 1−*T*, where transmittance *T* is obtained by normalizing the Fourier-transformed THz intensity spectrum of the Bi_2_Se_3_ micro-ribbon on sapphire substrate to that of bare substrate. Time-resolved plasmonic extinction dynamics under optical pump injection is measure by using ultrafast optical pump THz-probe spectroscopy. 1.55 eV (50 fs) pulses serve as an optical modulation pump. The pump (probe) spot size is 400 μm (300 μm).

### Plasmon–phonon interaction model analysis

Extinction coefficient arising from plasmon–phonon interaction can be described by the following model^20^,^46^;


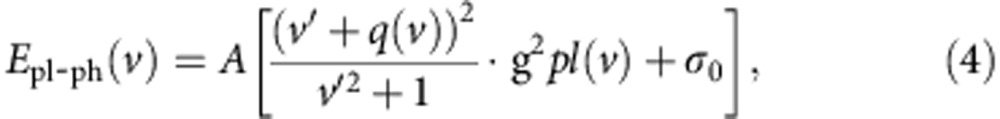


where 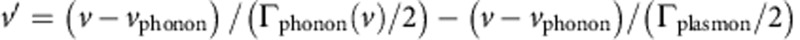 is the normalized frequency, 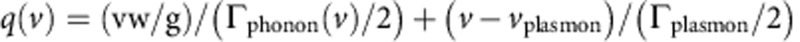 is the frequency-dependent Fano parameter, 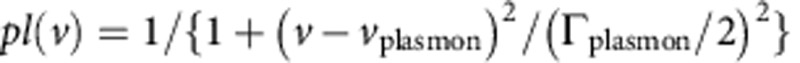 is the Lorentzian lineshape of the plasmon response, *σ*_0_ is the background absorption and *A* is the normalization constant^47^. Here, the parameter g (*w*) measures the coupling from ground to the bare plasmon (phonon) modes and *v* measures the plasmon–phonon interaction strength. The bare plasmon response is defined by*A*g^2^ *pl*(*v*). For the linewidth, while Γ_plasmon_ describes that of the non-interacting bare plasmon resonance, the phonon linewidth 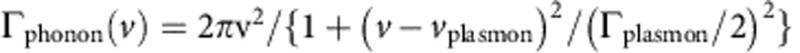 is considered only in the regime of plasmon–phonon interaction. This model regards the bare phonon mode as an ideal discrete state, whose linewidth measured in the unpatterned sample is indeed one order less than that of the plasmon resonance (see Supplementary Note 1).

## Additional information

**How to cite this article:** Sim, S. *et al.* Ultra-high modulation depth exceeding 2,400% in optically controlled topological surface plasmons. *Nat. Commun.* 6:8814 doi: 10.1038/ncomms9814 (2015).

## Supplementary Material

Supplementary Information Supplementary Figures 1-3, Supplementary Tables 1-3, Supplementary Notes 1-6 and Supplementary References.

## Figures and Tables

**Figure 1:**
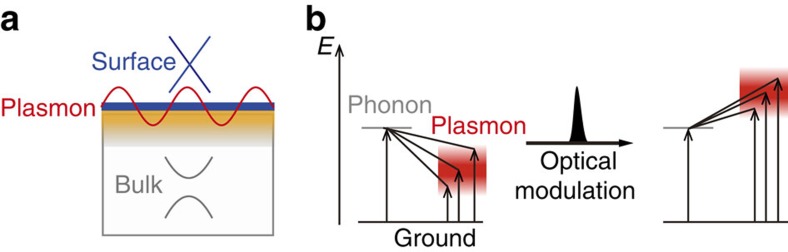
Schematics illustrating optical modulation of plasmon. (**a**) Coexistence of metallic surface states exhibiting gapless Dirac dispersion (blue region and X-shaped dispersion) and underlying normal gapped insulator (grey square and parabolic *E*-*k* dispersion) provides a novel platform for active modulation of the surface plasmon (red wave). Yellow-shaded region represents topologically trivial 2DEG, which also contributes to the collective charge oscillation. (**b**) Diagrams describing the optically modulated Fano-type interference between the narrow phonon state and the relatively broad plasmon mode. Upon optical excitation, the plasmon shift causes change in the THz transitions.

**Table 1:** Modulation depth and figure of merit in various photoexcited materials.

	**Modulation depth (%)**	**Pump fluence (mJ** **cm**^**−2**^**)**	**Figure of merit**
TI (this work)	2,400±200	0.045	50,000
Indium arsenide^5^	1,100	0.060	18,000
Graphene^10^	4	0.04	100
Gold^11^	3	0.5	6
Gold/silicon^3^	90	2.2	40
Aluminium/silica^4^	7.5	10	0.8

TI, topological insulator.
