# Supplementary material for: Ultra-high modulation depth exceeding 2,400% in optically controlled topological surface plasmons
Source: Nat Commun. 2015 Oct 30;6:8814. doi: 10.1038/ncomms9814 (PMC4640142; doi:10.1038/ncomms9814)
Supplement: Supplementary Information — Supplementary Figures 1-3, Supplementary Tables 1-3, Supplementary Notes 1-6 and Supplementary References. [file ncomms9814-s1.pdf]

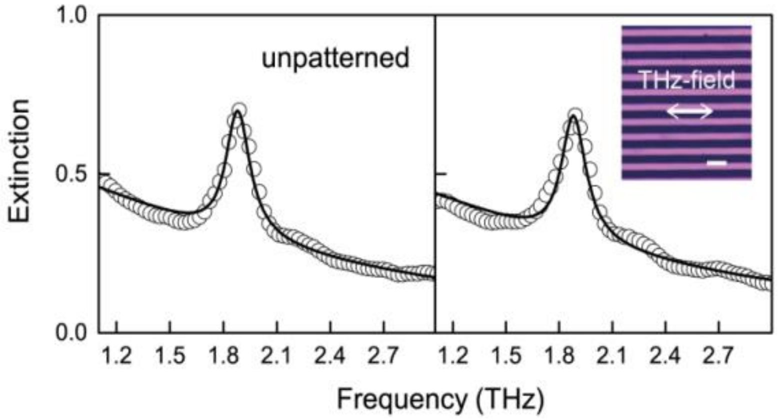

Supplementary Figure 1 | THz extinction spectra without optical pump injection. THz extinction spectra of unpatterned (black circles in the left panel) and patterned Bi_2Se_3 sample with parallel THz polarization to the ribbon axis (black circles in the right panel). Black solid lines are fits obtained by Drude-Lorentz model. Inset (in the right panel) shows an optical image of the patterned sample, which is same with the inset of Fig. 2 in the main text, except the white arrow illustrating THz polarization.

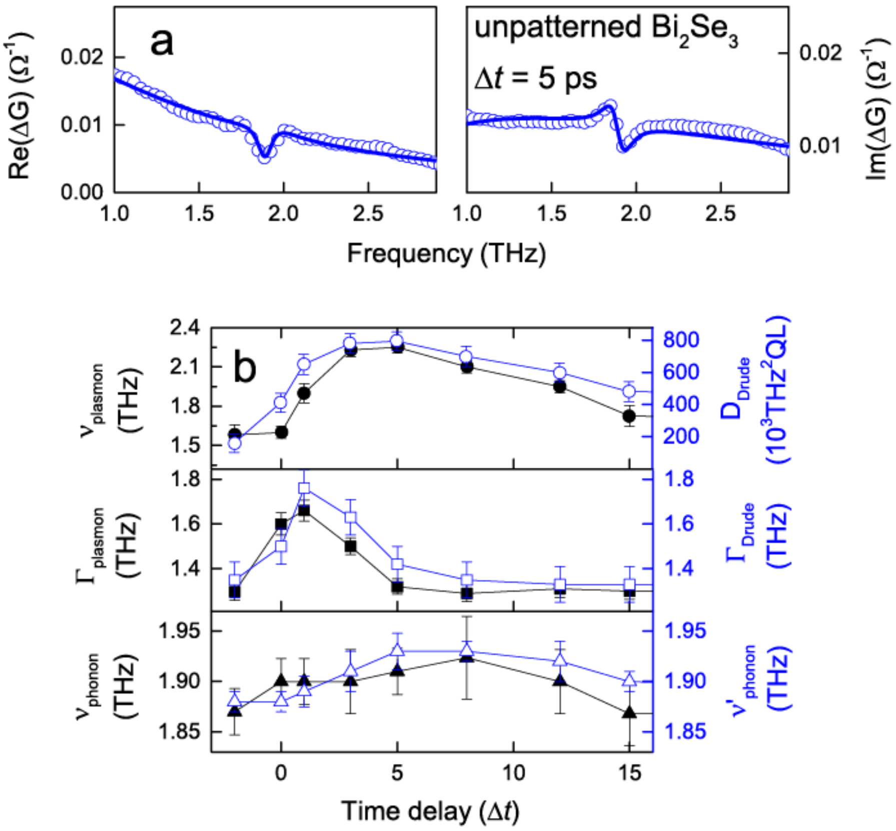

Supplementary Figure 2 | Time-resolved THz dynamics of unpatterned topological insulator Bi_2Se_3 thin film. (**a**) Complex differential sheet conductance ΔG spectra of an unpatterned topological insulator Bi_2Se_3 thin film (thickness = 100 QL) at Δ*t* = 5 ps, under 1.55 eV optical pump injection with fluence of 45 μJ cm^{-2}. Blue circles in the left (right) panel are real (imaginary) part of ΔG. Blue solid lines are Drude-Lorentz model fits (see Ref. 2 for the detailed fitting process). (**b**) Temporal evolution of the corresponding Drude-Lorentz model parameters is shown (blue symbols, right axis). For comparison, we simultaneously plot the plasmon-phonon interaction model parameters extracted by fitting the transient extinction spectra in Fig. 3b of the main text (black symbols, left axis). The error bars represent 95 % confidence intervals for the fitting parameters.

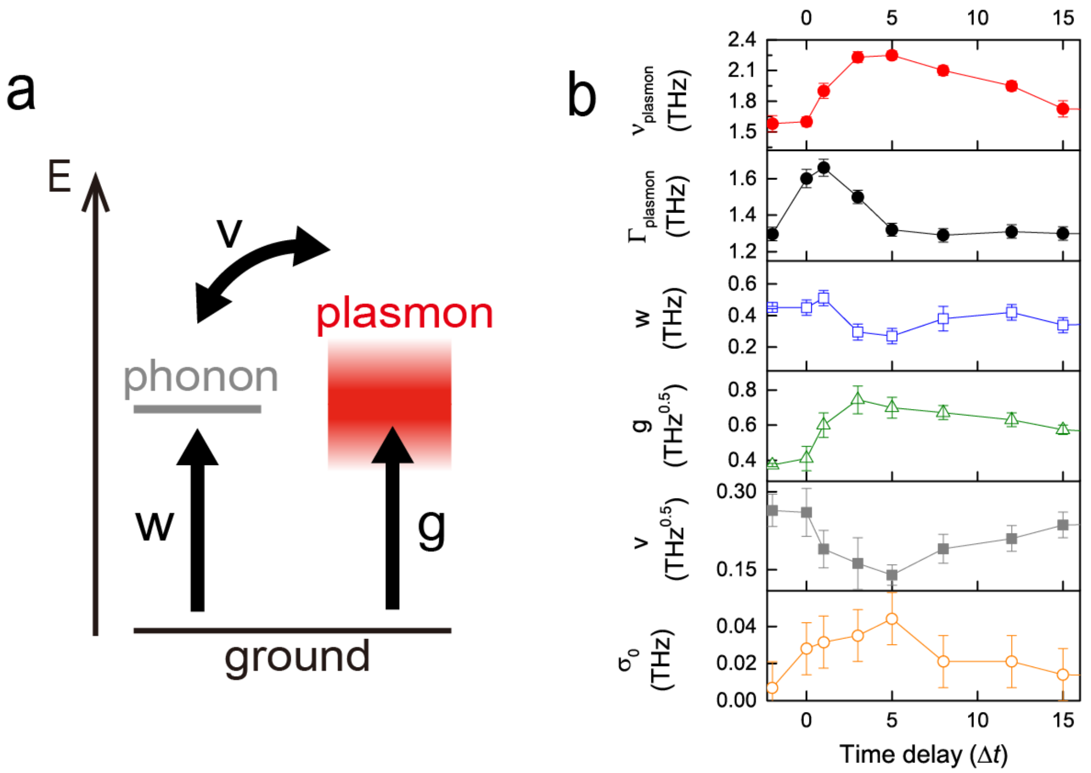

Supplementary Figure 3 | Details of plasmon dynamics. (**a**) Schematic diagram illustrating the plasmon-phonon interaction model. (**b**) Transient dynamics of the plasmon-phonon interaction model parameters extracted by fitting the extinction spectra in Fig. 3b of the main text. The error bars represent 95 % confidence intervals for the fitting parameters.

$\nu_{plasmon}$ (THz)	$\Gamma_{plasmon}$ (THz)	ν_{phonon} (THz)	w (THz)	g ($THz^{0.5}$)	v ($THz^{0.5}$)	σ_0 (THz)
1.58	1.29	1.87	0.45	0.37	0.26	0.007

Supplementary Table 1. Best fitting parameters of the plasmon-phonon interaction model on the extinction spectrum in the Fig. 2 of the main text. Details of the parameters are described in Method.

	D_{Drude} ($10^3 THz^2 QL$)	Γ_{Drude} (THz)	$SW_{Lorentz}$ ($10^3 THz^2 QL$)	$\Gamma_{Lorentz}$ (THz)	$\nu'_{Lorentz}$ (THz)
Unpatterned	160	1.30	60	0.120	1.89
Parallel THz polarization to the patterned sample	151	1.33	59	0.125	1.88

Supplementary Table 2. Best fitting parameters of the Drude-Lorentz model on the extinction spectra shown in the Fig S1 of the main text. For the Drude response, D_{Drude} (Γ_{Drude}) is the Drude spectral weight (linewidth). For the phonon Lorentz oscillator, $SW_{Lorentz}$ ($\Gamma_{Lorentz}$) is spectral weight (linewidth) and $\omega_{Lorentz}$ is the center frequency. Details of the fitting parameters are described in Ref. 2

	□plasmon (THz)	□plasmon (THz)	□phonon (THz)	w (THz)	g ($THz^{0.5}$)	v ($THz^{0.5}$)	σ_0 (THz)
without pump	1.53	1.80	1.90	0.41	0.37	0.26	0.011
with pump (Δt = 5 ps)	2.28	2.20	1.92	0.24	0.76	0.15	0.037

Supplementary Table 3. Best fitting parameters of the plasmon-phonon interaction model on the extinction spectrum at 300 K in the Fig. 5 of the main text.

Supplementary Note 1. THz extinction spectra without optical pump injection

In this section, we discuss the extinction responses of both patterned and unpatterned Bi_2Se_3 thin film samples without optical pump injection. As displayed in the Fig. 2 of the main text, the patterned sample exhibits the Dirac plasmon response with THz polarization perpendicular to the ribbon axis. The measured lineshape can be understood by the plasmon-phonon interaction model analysis. Corresponding fitting parameters in Supplementary Table 1 are well in accordance with the prior investigation[1]. The unpatterned sample, unlike the plasmonic response, shows a simple Drude response of Dirac electrons, whose featureless shape overlaps with the narrow ~1.9 THz phonon Lorentz oscillator (see Supplementary Figure 1a). It reveals that the THz irradiation cannot be coupled to the collective charge oscillations due to their momentum mismatch. For the same reason, the patterned micro-ribbon sample also exhibits the Drude-Lorentz response when the THz polarization is parallel to the ribbon axis, as shown in Supplementary Figure 1b (see Ref. 2 for the detailed description of the Drude-Lorentz model analysis). Corresponding fitting parameters, displayed in Supplementary Table 2, agree with prior studies.

Supplementary Note 2. Time-resolved THz dynamics of unpatterned Bi_2Se_3 thin film

To more deeply understand the time-resolved plasmon responses of the patterned sample, it is instructive to compare them with the intrinsic THz dynamics of bare Bi_2Se_3 sample. While the extinction measurements are suitable for characterizing the spectral response of plasmon-phonon interaction, it is known that the transient differential complex sheet conductance (ΔG) provides a powerful tool for measuring the pump-induced subtle changes in the Drude response of bare TI. For this reason, following ref. 2, we measure ΔG of the unpatterned topological insulator Bi_2Se_3 thin film sample with thickness of 100 QL by using time-resolved optical-pump THz-probe spectroscopy. The 1.55 eV (50 fs) pulses with fluence of 45 μJcm^{-2} serve as the pump. Figure S2a shows measured ΔG spectra (blue circles) and the corresponding Drude-Lorentz fits (blue lines) at Δt = 5 ps, which agree with those in Ref. 2 (see Ref. 2 for details of analysis). In Supplementary Figure 2b, we display the

time-resolved Drude-Lorentz model parameters; Drude spectral weight D_{Drude} (blue circles in the top panel), Drude scattering rate Γ_{Drude} (blue squares in the middle panel) and optical phonon centers (blue triangles in the bottom panel) are shown. For comparison, we simultaneously plot the plasmonic parameters extracted from the time-resolved extinction of the patterned sample through fitting with the plasmon-phonon interaction model (Fig. 3b in the main text).

We now discuss the dynamics of these parameters. First, the transient D_{Drude} closely follows the dynamics of plasmon center frequency (black circles in the top panel of Supplementary Figure 2b). Because D_{Drude} measures the pump-generated carrier population, the result supports the attribution of the plasmon shift to the increased carrier density in the main text. Second, we see that the dynamics of plasmon linewidth $\Gamma_{plasmon}$ (black squares in the middle panel of Supplementary Figure 2b) closely follows that of Γ_{Drude}. This observation suggests that the transient plasmon damping is mainly determined by the electronic scattering rate in the photoexcited regime. Also notable is that the decay dynamics of Γ_{Drude} is very fast compared to the transient D_{Drude}. It can be understood by different origins of these parameters; while the dynamics of Γ_{Drude} arises from the pump-induced change in the scattering rate of the surface electrons, the main origin of transient D_{Drude} dynamics is the pump-generated carrier populations in the bulk states. Finally, we compare the dynamics of phonon frequency extracted from the Drude-Lorentz fit to the ΔG response of bare Bi_2Se_3 (black triangles in the bottom of Supplementary Figure 2b) with that from the plasmon-phonon interaction model fits to extinction spectra of patterned sample (blue triangles in the bottom of Supplementary Figure 2b). They are very similar with each other, justifying the analysis of the transient plasmon response in the main text.

Supplementary Note 3. Details of the plasmon frequency formula

In this section, we briefly discuss the origin of the pre-factor '4' of E_F^{2DEG} in Eq. (2) in the main text.

According to the ref. 4 and 5, the frequency of collective charge oscillation ω_p in a general electronic

system can be defined by,

$$\omega_{\mathrm{p}}^2 = \chi_{jj} q^2 \upsilon_q, \qquad (1)$$

where q is the wavevector and $\upsilon_q = e^2 / (2\varepsilon_0 \varepsilon\, q)$ is the Coulomb interaction in two-dimension. The local longitudinal current response (χ_{jj}) can be expressed as a function of the chemical potential (μ),

$$\chi_{jj} = \frac{g_\upsilon g_s \xi \mu}{4\pi\hbar^2}, \qquad (2)$$

where g_{v}, g_{s} are the valley- and spin- degeneracy, respectively. For the Dirac surface,

$g_{\mathrm{s}} = g_{\mathrm{v}} = 1$, but $g_{\mathrm{s}} = 2$, $g_{\mathrm{v}} = 1$ for the spin-degenerated 2DEG state. The parameter ξ is related to the energy-momentum dispersion $E(k) \propto |k|^{\xi}$; while $\xi = 1$ for the linear Dirac surface state, ξ is 2 for the 2DEG. Thus, compared to the Dirac state, the longitudinal current response of 2DEG state is four times larger, resulting in the pre-factor 4 of $E_{\mathrm{F}}^{\mathrm{2DEG}}$ in the Eq. (2) in the main text.

Supplementary Note 4. Details of plasmon dynamics

In this section, we discuss details of time-resolved plasmon dynamics in Fig. 3b of the main text. The plasmon-phonon interaction model has several coupling parameters as well as bare phonon and plasmon response (see Method for the detailed description of this model). Supplementary Figure 3a illustrates the light-induced coupling between each states and the corresponding parameters; w and g indicates the coupling of ground state to phonon and plasmon, respectively, and parameter v describes the strength of interaction between these two states[1,6]. To understand the plasmon dynamics, we discuss the change in these couplings under optical injection.

In Supplementary Figure 3b, we plot the time-resolved transients of parameters for the plasmon-phonon interaction model. Here, we plot again the dynamics of bare plasmon frequency (ν_{plasmon}, red

circles) and linewidth ($\Gamma_{plasmon}$, black circles) in the main text for clear comparison. First, while the transition from ground to phonon mode (w, blue squares) does not show significant changes upon photoexcitation, the transient coupling between the ground and the plasmon (g, green triangles) exhibits meaningful dynamics, which essentially follow the temporal evolution of the plasmon resonance $\nu_{plasmon}$. Both g and $\nu_{plasmon}$ show their maximum at Δt = 5 ps, which can be understood by the pump-induced increase of the surface carriers contributing to the collective charge oscillation. Second, the coupling between phonon and plasmon is largely decreased upon photoexcitation (v, gray squares). Notable is that it reaches its minimum at Δt = 5 ps, at which $\nu_{plasmon}$ has a peak value. This coincidence may be explained by the origin of plasmon shift. As discussed in the main text, without optical injection, the main species responsible for the plasmon excitation is the TSS electrons, which exhibit a strong coupling with the 1.9 THz phonon. In contrast, 2DEG dominates the plasmon excitation under optical injection, which may account for the observed decrease in the plasmon-phonon interaction (v) because the reported 2DEG-phonon coupling constant[7] is much smaller than that for the TSS electrons[8]. However, more theoretical studies are needed for complete understanding. Finally, we plot the transient background absorption (σ_0, yellow circles), which has its maximum at Δt = 5 ps like transient $\nu_{plasmon}$. This behavior is quite reasonable since both σ_0 and $\nu_{plasmon}$ should follow the population dynamics of pump-generated carriers.

Supplementary Note 5. Bulk contribution to the plasmon resonance

Generally, the light-coupled surface plasmon excitations are considered in two different regimes: collective oscillations of the confined 2D charges and surface excitations of the unconfined 3D charges[9,10]. In the main text, while we have attributed the observed plasmon resonance in the TI to the excitations of ‘confined’ charges (TSS and 2DEG) both with and without optical injections, it needs to check other possible contributions, such as from the unconfined 3D carriers to the plasmon resonance, because the optical pump can generate a considerably large amount of bulk carriers (~order of 10^{19}

cm^{-3}). Note that, in Ref. 1, the contributions from the unconfined 3D charges are excluded, maybe due to their low density. Our calculations show that the surface plasmon frequency of the unconfined 3D carriers (generated by optical pump) is far beyond the 2DEG plasmon. Although there is no significant difference between the 2DEG thickness (~4 nm) and the optical pump penetration depth (~20 nm), the corresponding plasmon frequencies exhibit a large difference because their dimensionalities are different (i.e. 2D and 3D, respectively). Unlike 2DEG, the three-dimensional bulk carriers are not spatially confined in the band bending region, even if the pump-generated carriers are transiently concentrated within 20 nm from the surface. Thus, we have used two different formula to calculate the plasmon frequencies of the confined 2D (TSS and 2DEG) and the unconfined 3D bulk charges, respectively. That is why the calculated plasmon frequencies are not simply proportional to the spatial thickness of the charge distributions.

Supplementary Note 6. Dissipation pathways of excess energy injected by the optical control pulse

In the main text, we have shown that the large modulation of topological surface plasmon response can be achieved by injecting optical control pulse. Since the photon energy of the optical control pulse (1.55 eV) is much larger than the bulk band-gap of Bi_2Se_3 (~250 meV), it is instructive to discuss the dissipation pathways of the excess energy injected by the control pulse. In this section, we briefly discuss the dynamic pathways of the excess energy.

After the photoexcitation, the injected photons are initially absorbed by electrons, producing non-thermal electrons and holes with high energies in a conduction and valence band, respectively. Then, a fast carrier-carrier scattering takes place within 200 fs, leading to the increased effective carrier temperature, which can be described by the so called “hot quasi-Fermi distributions”[11]. Consequently, the carrier scattering rate strongly increases[2], which gives rise to the broadening of the plasmon response because the plasmon damping rate directly depends on the carrier scattering rate. This

scenario can explain the initial, fast rise of the plasmon linewidth (Fig. 3d in the main text). Next, the excess carrier energies are transferred to the lattice via the carrier-phonon scattering, whose time-scales are typically on the order of 1-10 picoseconds[2,12–15]. As a result of this process, the lattice temperature is increased, and consequently the optical phonon mode near 2 THz slightly stiffens[14,15]. In our measurements, we have observed that the phonon frequency increases upon photoexcitation, which reaches its maximum near Δt = 5-10 ps (see the change in the position of gray vertical lines in Fig. 3b of the main text and the bottom panel of Supplementary Figure 2b). It directly indicates the time-scale of energy transfer from the hot carries to the lattice; the excess thermal energy is eventually transferred to the substrate. In addition to this carrier-phonon scattering, the system recovers its thermodynamic equilibrium via various relaxation pathways, such as hot carrier diffusion[16,17], radiative and non-radiative recombination[18,19] and defect scattering[18]. These processes are possibly related to the observed recovery dynamics of the plasmon response.

Supplementary References

1. Di Pietro, P. *et al.* Observation of Dirac plasmons in a topological insulator. *Nat. Nanotechnol.* **8,** 556–560 (2013).

2. Sim, S. *et al.* Ultrafast terahertz dynamics of hot Dirac-electron surface scattering in the topological insulator Bi_2Se_3. *Phys. Rev. B* **89,** 165137 (2014).

3. Okada, Y. & Madhavan, V. Topological insulators: plasmons at the surface. *Nat. Nanotechnol.* **8,** 541–542 (2013).

4. Stauber, T., Gómez-Santos, G. & Brey, L. Spin-charge separation of plasmonic excitations in thin topological insulators. *Phys. Rev. B* **88,** 205427 (2013).

5. Stauber, T. Plasmonics in Dirac systems: from graphene to topological insulators. *J. Phys. Condens. Matter* **26,** 123201 (2014).

6. Giannini, V., Francescato, Y., Amrania, H., Phillips, C. C. & Maier, S. A. Fano resonances in nanoscale plasmonic systems : a parameter-free modeling approach. *Nano Lett.* **11,** 2835–2840 (2011).

7. Barreto, L. *et al.* Electron-phonon coupling in the two-dimensional electron gas on Bi_2Se_3. *Phys. status solidi - Rapid Res. Lett.* **7,** 136–138 (2013).

8. Zhu, X. *et al.* Electron-phonon coupling on the surface of the topological insulator Bi_2Se_3 determined from surface-phonon dispersion measurements. *Phys. Rev. Lett.* **108,** 185501 (2012).

9. Yoon, H., Yeung, K. Y. M., Kim, P. & Ham, D. Plasmonics with Two-Dimensional Conductors. *Phil. Trans. R. Soc. Lond. A* **372,** 20130104 (2014).

10. Park, S. J. & Palmer, R. E. Acoustic Plasmon on the Au(111) Surface. *Phys. Rev. Lett.* **105,** 016801 (2010).

11. Crepaldi, A. *et al.* Ultrafast photodoping and effective Fermi-Dirac distribution of the Dirac particles in Bi_2Se_3. *Phys. Rev. B* **86,** 205133 (2012).

12. Wang, Y. H. *et al.* Measurement of intrinsic Dirac fermion cooling on the surface of the topological insulator Bi_2Se_3 using time-resolved and angle-resolved photoemission spectroscopy. *Phys. Rev. Lett.* **109,** 127401 (2012).

13. Glinka, Y. D. *et al.* Ultrafast carrier dynamics in thin-films of the topological insulator Bi_2Se_3. *Appl. Phys. Lett.* **103,** 151903 (2013).

14. Valdés Aguilar, R. *et al.* Time-resolved terahertz dynamics in thin films of the topological insulator Bi_2Se_3. *Appl. Phys. Lett.* **106,** 011901 (2015).

15. Sim, S., Koirala, N., Brahlek, M., Park, J. & Cha, S. Tunable Fano quantum-interference dynamics using topological phase transition in $(Bi_{1-x}In_x)_2Se_3$. *Phys. Rev. B* **91,** 235438 (2015).

16. Sobota, J. A. *et al.* Ultrafast optical excitation of a persistent surface-state population in the topological insulator Bi_2Se_3. *Phys. Rev. Lett.* **108,** 117403 (2012).

17. Kumar, N. *et al.* Spatially resolved femtosecond pump-probe study of topological insulator Bi_2Se_3. *Phys. Rev. B* **83,** 235306 (2011).

18. Glinka, Y. D., Babakiray, S., Johnson, T. A., Holcomb, M. B. & Lederman, D. Effect of carrier recombination on ultrafast dynamics in thin films of the topological insulator Bi_2Se_3. *Appl. Phys. Lett.* **105,** 171905 (2014).

19. Onishi, Y. *et al.* Ultrafast carrier relaxation through Auger recombination in the topological insulator $Bi_{1.5}Sb_{0.5}Te_{1.7}Se_{1.3}$. *Phys. Rev. B* **91,** 085306 (2015).
